# Metabolomic Studies of Tissue Injury in Nonhuman Primates Exposed to Gamma-Radiation

**DOI:** 10.3390/ijms20133360

**Published:** 2019-07-09

**Authors:** Amrita K. Cheema, Khyati Y. Mehta, Meena U. Rajagopal, Stephen Y. Wise, Oluseyi O. Fatanmi, Vijay K. Singh

**Affiliations:** 1 Department of Oncology, Lombardi Comprehensive Cancer Center, Georgetown University Medical Center, Washington, DC 20001, USA; 2 Department of Biochemistry, Molecular and Cellular Biology, Georgetown University Medical Center, Washington, DC 20001, USA; 3 Department of Pharmacology and Molecular Therapeutics, F. Edward Hébert School of Medicine, USUHS, Bethesda, MD 20814, USA; 4 Scientific Research Department, Armed Forces Radiobiology Research Institute, USUHS, Bethesda, MD 20814, USA

**Keywords:** acute radiation syndrome, biomarker, gamma-radiation, lipidomes, metabolites, nonhuman primates, tissue

## Abstract

Exposure to ionizing radiation induces a complex cascade of systemic and tissue-specific responses that lead to functional impairment over time in the surviving population. However, due to the lack of predictive biomarkers of tissue injury, current methods for the management of survivors of radiation exposure episodes involve monitoring of individuals over time for the development of adverse clinical symptoms and death. Herein, we report on changes in metabolomic and lipidomic profiles in multiple tissues of nonhuman primates (NHPs) that were exposed to a single dose of 7.2 Gy whole-body ^60^Co γ-radiation that either survived or succumbed to radiation toxicities over a 60-day period. This study involved the delineation of the radiation effects in the liver, kidney, jejunum, heart, lung, and spleen. We found robust metabolic changes in the kidney and liver and modest changes in other tissue types at the 60-day time point in a cohort of NHPs. Remarkably, we found significant elevation of long-chain acylcarnitines in animals that were exposed to radiation across multiple tissue types underscoring the role of this class of metabolites as a generic indicator of radiation-induced normal tissue injury. These studies underscore the utility of a metabolomics approach for delineating anticipatory biomarkers of exposure to ionizing radiation.

## 1. Introduction

Nuclear accidents, such as Chernobyl and Fukushima–Daiichi, and deliberate radiological events by terrorists have been at the forefront of emergency response planning [1,2,3]. The threat of terrorism or military action has escalated the possible use of radiological or nuclear weapons. Further, the atomic bombings of Hiroshima and Nagasaki have demonstrated the power of nuclear weapons to cause harm to humans through morbidity, mortality, and long-term effects arising from radiation exposure. The detonation of an improvised nuclear device (IND) or a radiological dispersal device (RDD) requires an immediate assessment of exposed victims for the absorbed radiation dose. While an RDD will expose individuals to relatively low levels of radiation, an IND can lead to significant exposure and death to thousands of people [4]. Protection of citizens from national health security threats continues to be a high priority for the government.

Significant efforts have been made to develop sensitive methods for radiation exposure dose assessment and medical countermeasure efficacy. Complete blood count (CBC), dicentric measurements, premature chromosome condensation (PCC), gene expression, γ-H2AX, micronucleus assay, protein biomarkers, metabolic biomarkers, and electron paramagnetic resonance (EPR) or optically stimulated luminescence (OSL) of teeth have been used as some of the methods for identifying and validating biomarkers [5,6,7]. However, some of these assays require a long time and highly trained manpower to perform the assays and interpret the results. Radiation biomarkers should be able to accurately classify individuals as having >2 Gy of exposure. A dose of >2 Gy would lead to acute radiation syndrome (ARS) that can lead to death without appropriate medical intervention. A dose of <2 Gy, specifically 0.75–1 Gy, can also require treatment, although attention for those individuals may be less of a priority.

For first responders and military personnel, personal dosimetry may provide an accurate estimation of the external exposure and banked biological samples may serve as a reference for biodosimetry, which is not applicable to the general population. Though classical cytogenetic techniques are the gold standard for radiation dose assessment, as stated above, these assays are time-consuming, laborious, and require well-trained staff. One promising approach for biomarker identification is metabolomics, which allows rapid, qualitative, and quantitative assessment of small molecules of <1 kDa in tissues and biofluids. In addition, lipidomics, a comprehensive assessment of relative changes in endogenous levels of lipids, is considered a component of metabolomics analyses. Global molecular phenotyping approaches allow for the full scanning of the metabolome and pattern identification according to pathway interactions, whereas targeted approaches can be more quantitative and concentrate on specific metabolites or perturbations along a metabolic pathway. Metabolomics of high-dose radiation exposure has provided a highly revealing glimpse of metabolic dysregulation [8,9,10]. Biomarkers in both tissue and biofluid samples from mouse, rat, minipig, nonhuman primate (NHP), and humans have offered the basis for the determination of a radiation signature to assess the need for medical intervention [11]. Biomarkers of tissue-specific injury will be informative for treatment and for the future risk of delayed effects of radiation exposure. To date, only citrulline has been well identified through metabolomics as a reliable tissue-specific biomarker [12].

Here, we have performed a systemic study to delineate the metabolic profiles in heart, lung, spleen, jejunum, liver, and kidney of NHPs exposed to 7.2 Gy whole-body ^60^Co γ-radiation. Our results, for the first time, demonstrate that major metabolic organs such as liver and kidney show changes in glycerophospholipid metabolism, amino acid, and sugar metabolism, as well as fatty acid metabolism. Additionally, for the first time, we report on the elevation of long-chain acylcarnitines in the irradiated cohort with a high correlation in the heart of the survivor, as well as with the decedent cohort of NHPs. These findings show that exposure to ionizing radiation causes long-term changes in metabolism that can be used to monitor tissue damage.

## 2. Results

### 2.1. Untargeted Metabolomics Analysis Identifies Changes in the Tissue Metabolome of the NHPs Exposed to Radiation

**Figure 1:**
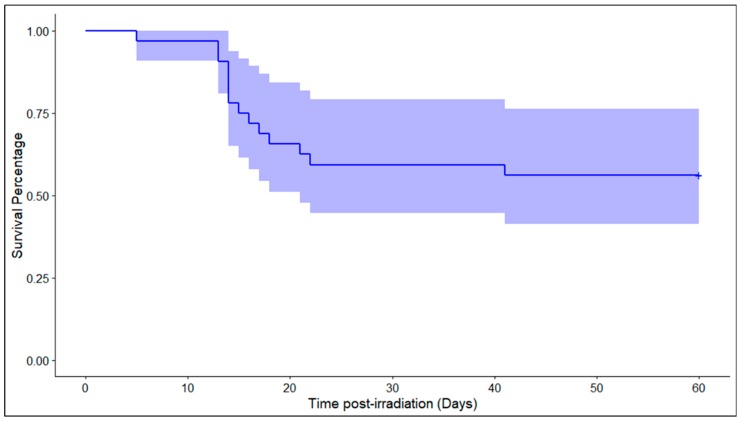
Percent survival for the nonhuman primate (NHP) study cohort over the course of 60 days of the follow-up period.

In order to study changes in tissue profiles of NHPs exposed to a lethal dose of radiation, we followed the survival patterns of an NHP for 60 days subsequent to whole-body irradiation. As shown in Figure 1, the survival pattern of individual NHPs receiving a single dose of radiation compared with healthy animals showed significant mortality (these animals are referred to as non-survivors hereafter). We harvested tissue from healthy, as well as the irradiated NHPs for metabolomic and lipidomic analyses of six tissue types including liver, kidney, heart, jejunum, spleen, and lung. We asked if there were differences in the molecular profiles of the irradiated cohort as compared to the healthy animals, as well as the effect of irradiation in decedents as compared to the surviving cohort.

Pre-processing of the raw LC-MS data through XCMS software identified a total of 3128 features in electrospray ionization (ESI) positive mode and 2725 in ESI negative mode. Initially, each NHP was labeled at ‘healthy’ or ‘irradiated’. The irradiated group was further split into two groups based on if they survived to the 60-day time point after radiation as ‘survivor’ and if they died at any point prior as ‘non-survivor’ that were used for statistical analysis. ANOVA comparison across all the three groups was performed followed by the following binary t-test: healthy vs. survivor, healthy vs. non-survivor, and survivor vs. non-survivor. The significant *m*/*z* values were then subjected to an accurate mass-based database search using CEU-Mass Mediator, the Human Metabolome Database (HMDB), and the Metlin for identification. After identification, biological endogenous metabolites were separated and verified using tandem mass spectrometry. ANOVA analyses yielded a maximum number of altered metabolites (as shown within the parenthesis) which were observed from kidney (115 features) and liver (71) tissue types, while jejunum (24), spleen (10), lung (5), and heart (1) tissues showed only moderate changes in the metabolite levels, and binary comparisons showed robust changes in all tissue types. A set of discriminating metabolites separating the healthy and irradiated groups were identified and tabulated (Supplementary Table S1).

### 2.2. Metabolite Alterations in the Kidney and Liver Profiles as Indicators of Radiation-Induced Tissue Damage

Liver and kidney tissue samples from 40 NHPs were subjected to untargeted metabolomics analysis using Waters QToF. A total of 3128 and 2725 features were detected in positive and negative electrospray ionization modes, respectively. The identity of significantly altered metabolites was confirmed by tandem mass spectrometry.

**Figure 2:**
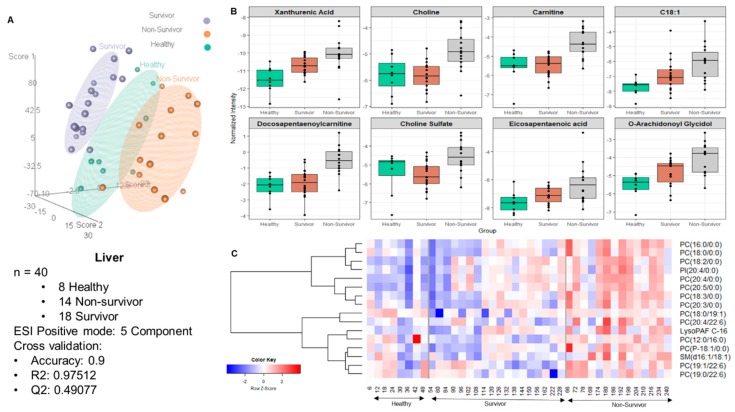
Exposure to ionizing radiation leads to robust changes in liver metabolomic and lipidomic profiles. Panel (**A**): Three dimensional PLS-DA plot showing separation of healthy NHPs (N = 8) from those who either survived (N = 18) or succumbed to radiation-induced (N = 14) tissue injury within 60 days. The prediction accuracy for 100 permutations yielded a p-value of 0.04. Panels (**B** and **C**): Relative abundance of significantly dysregulated metabolites and lipids in the three study groups, respectively.

**Figure 3:**
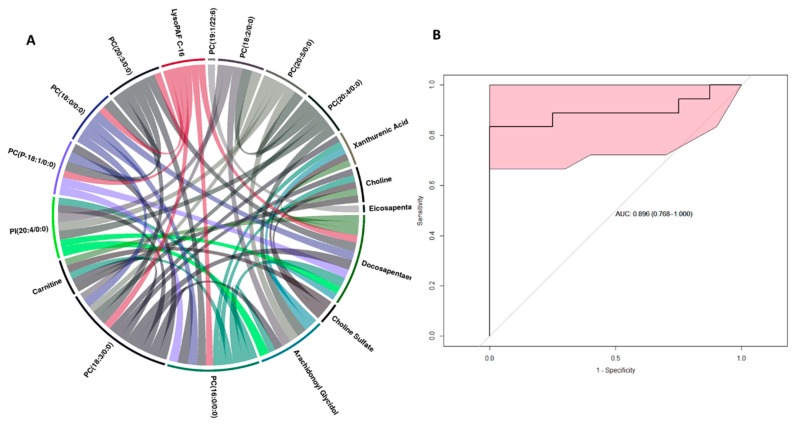
Metabolite correlates of radiation response in NHP liver. Panel A. Circos plot visualization of Spearman correlation values between 18 top cut-off point p-value < 1 × 10^−30^. Panel B. The ROC curve with a six-metabolite panel predictive of post-irradiation survival in NHPs liver. The classification algorithm showed sensitivity = 0.833, specificity= 0.875, AUC: 0.869, and a predictive accuracy of 0.846.

The score plots for liver tissue data (Figure 2, Panel A) revealed significant differences in metabolite profiles between healthy and non-survivors. Cross-validation of the PLS-DA (partial least square discriminant analysis) model yielded R^2^ = 0.9751 and Q^2^ = 0.4908 in the liver samples in positive mode, suggesting good separation of healthy NHPs (N = 8) from those who succumbed to radiation-induced tissue injury (N = 14) within 60 days. The liver metabolite profile of NHPs that survived radiation, was seen to overlap among the healthy and non-survivors. These results suggest that NHPs exposed to whole-body radiation induced significant changes in the liver metabolome. Next, we performed ANOVA analyses to find metabolites that significantly changed after exposure to radiation. Multiple metabolites were significantly altered in the liver of NHPs that were exposed to radiation compared to the healthy cohort. Of particular interest, were the differences in the levels of xanthurenic acid, choline, carnitine, C18:1, decosopentanoylcarnitine, choline sulfate, O-arachidonoyl glycidol, and eicosapentaenoic acid (Figure 2, Panel B). Interestingly, alterations in the levels of these metabolites followed a pattern wherein they were either incrementally decreased or increased from healthy to survivors to non-survivors suggesting a correlation with survival time post-irradiation. Additionally, hierarchical clustering revealed an alteration in the levels of lipids in the healthy NHP liver compared to NHPs exposed to radiation (Figure 2, panel C). Next, we asked if there was a correlation between the metabolite abundance pattern and survival in the NHP cohort. For this purpose, we performed Spearman correlation analysis that was visualized as a circus plot (Figure 3, Panel A) and found PC(18:3/0:0) PC(16:0/0:0) to be highly correlated with survival (p-value cut-off ≤ 1 × 10^−30^). The ROC (receiver operator characteristic) analysis using 10-fold cross-validation augmented the selection of a four-metabolite panel predictive of post-irradiation survival in NHPs with 89% accuracy (Figure 3, Panel B). The metabolite comprising this panel included xanthurenic acid, C18:1, Carnitine, and PC (18:3/0:0).

**Figure 4:**
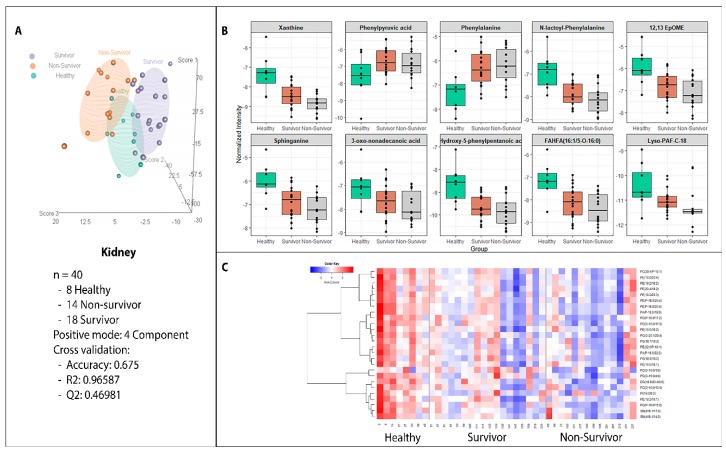
Metabolomic and Lipidomic profiles of kidney in NHPs pre and post-irradiation. Panel A: Three dimensional PLS-DA plot showing separation of healthy NHPs (N = 8) from those who either survived (N = 18) or succumbed to radiation-induced (N = 14) tissue injury within 60 days.Prediction accuracy for 100 permutations yielded a p-value of 0.07. Panels B and C: the relative abundance of significantly dysregulated metabolites and lipids in the threes study groups, respectively.

**Figure 5:**
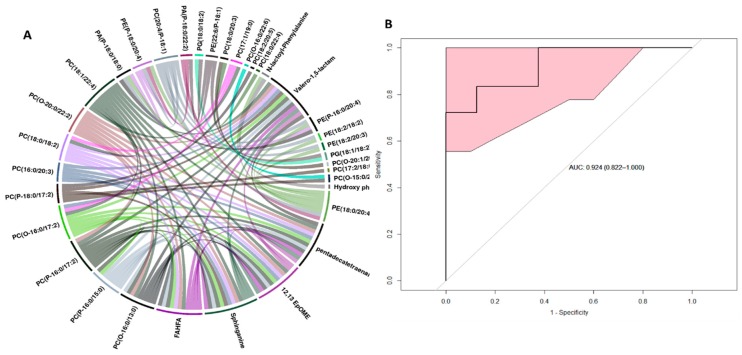
Metabolite correlates of radiation response in NHP kidney. Panel (**A**) Circos plot of Spearman correlation values between 18 top cut-off point p-value < 1 × 10^−30^. Panel (**B**) the ROC curve with a six-metabolite panel predictive of post-irradiation survival in NHP kidney. The classification algorithm showed sensitivity = 0.833, specificity = 0.875, AUC: 0.924 and a predictive accuracy of 0.849.

In addition to the liver, profiling of the kidney showed a maximum number of altered metabolites between healthy and irradiated groups. As seen in Figure 4, panels A and B, hierarchical clustering and three dimensional PLS-DA plot showed separation of healthy NHPs (N = 8) from those who either survived (N = 18) or succumbed to radiation-induced (N = 14) tissue injury within 60 days (R^2^ = 0.9660 and Q^2^ = 0.4698). Of the 44 metabolites that were significantly altered due to radiation exposure, xanthine, phenylpuruvic acid, phenylalanine, N-lactoyl phenylalanine, sphinganine, 3-oxo nonadecanoic acid, 3-hydroxy 5-phenylpentanoic acid, FAHFA (fatty acid hydroxyl fatty acid), lyso PAF-C18, and 12,13 EpOME were significantly altered between the kidney from the healthy NHPs versus irradiated NHPs (Figure 4, panel B). In addition, we found significant dyslipidemia that was progressive between healthy, survivor, and the non-survivor NHPs (Figure 4, panel C). These included alterations in glycerophospholipids, glycerophosphoethanolamines, and sphingomyelins. A Spearman correlation analysis showed that PC(18:1/22:4), PC(18:0/20:3), PC(17:1/19:0), and PC(16:0/20:3) in kidney were the most correlated metabolites with irradiated surviving NHPs (Figure 5, Panel A). Finally, a six-metabolite panel that included PE 16:0/18:1, SM (16:1/17:0), xanthine, N-lactoyl-phenylalanine, Sphinganine, PE (16:0/20:4) was able to discriminate healthy and surviving NHPs by >90% predictive accuracy (Figure 5, Panel B).

### 2.3. Generic Markers of Ionizing Radiation Exposure Across Multiple Tissue Types

**Figure 6:**
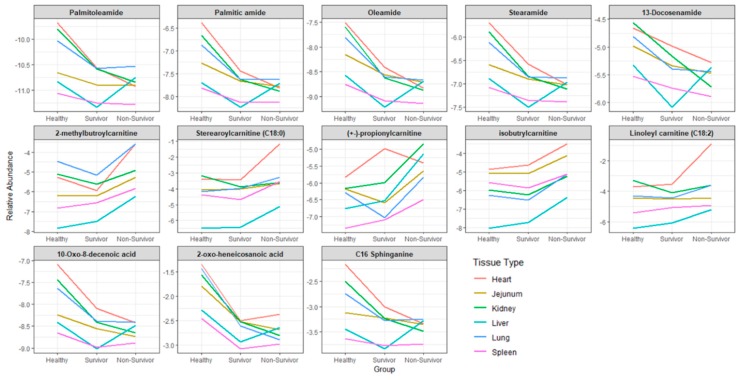
Overlapping patterns of metabolite abundance in the three NHP study groups across different tissue types. The Y-axis shows mean values for the log-transformed and Pareto scaled relative abundances.

Although kidney and liver tissues types showed a maximum number of altered metabolites, exposure to radiation generated a modest metabolic response in other organs like heart, jejunum, lung, and spleen. Broadly, the altered metabolites across tissue type could be classified as fatty acid amides, long chain acylcarnitines, and oxidized fatty acids. A trend plot analysis showed a progressive pattern of abundance between the different study groups. For example, the endogenous levels of fatty acid amides were considerably decreased in the survivors and non-survivors (Figure 6). On the other hand, levels of most of the acylcarnitines were found to be increased in the non-survivors compared to healthy NHPs, though the levels slightly decreased in the survivors. The levels of oxo-fatty acids also showed a relative decrease in the radiation-exposed NHPs as compared to the healthy groups. Overall, radiation caused alterations in the levels of metabolites of other soft tissues like heart, jejunum, lung, and spleen with the maximum effect observed in the kidney and liver at 60 days’ post-irradiation.

## 3. Discussion

Accidental exposures, nuclear accidents, and elevated threats of terrorism with the potential detonation of an IND or a RDD in a metropolitan city have led to an increased need for the rapid assessment of exposure to different radiation qualities and dose. The qualitative and quantitative assessment of small molecules in a given biological specimen, metabolomics, has emerged as a promising technology to allow for rapid determination of an individual’s exposure level and metabolic phenotype. Advancements in mass spectrometry techniques have led to targeted and untargeted methods identifying biomarkers of radiation exposure much earlier than the appearance of gross clinical symptoms. Moreover, unlike ARS, which occurs rapidly after irradiation, there is an adequate latent window for predicting delayed effects of acute radiation exposure (DEARE), increasing the feasibility of using biomarkers. Thus far, no biodosimetry approach has been cleared by the U.S. Food and Drug Administration (FDA) for use in triage or as a diagnostic tool. Moreover, most of the research efforts have focused on the delineation of circulating biomarkers detectable in the first 48 h after radiation exposure [13,14]; as such, there is a paucity of studies focused on circulating biomarkers that indicate or predict the development of delayed radiation injury in organs such as the kidney, heart, and brain, after exposure to both high- and low-LET (linear energy of transfer) radiation [15,16].

We found that the liver and kidney showed most robust changes in metabolic and lipid profiles at the 60-day time point in survivors. For example, we found changes in arachidonic acid metabolism, carnitine, and choline metabolism in the liver tissue post-irradiation. Changes in arachidonic acid metabolism are suggestive of radiation-induced tissue injury pertaining to fibrosis that manifests as a radiation late effect [17]. Previous studies have shown the release of choline and carnitine due to significant hepatic damage resulting from exposure to high doses of ionizing radiation [18]. We also found dyslipidemia in the liver tissue that is consistent with reports from other groups for circulating biomarkers of radiation exposure [19,20]. Remarkably, dyslipidemia was more severe in the non-survivors as compared to the survivors and the healthy NHPs indicating a strong correlation with overall survival following exposure to radiation. Radiation-induced dyslipidemia could potentially be a consequence of increased lipid peroxidation. Lipid peroxidation, specifically the peroxidation of polyunsaturated fatty acids after exposure to radiation, is well documented by several studies that have identified cellular damage at various levels including DNA, proteins, and membrane lipids [21,22]. FAs (fatty acids) are vital endogenous molecules for cellular energy metabolism, and the decrease in their levels following irradiation is most likely a protective mechanism of these cells to meet their energy demands under this hypoxic condition. In addition, we found a significant increase in Lyso PAF C-16, which has been reported to accumulate as a result of ionizing radiation-mediated oxidation of phospholipids leading to tissue damage and inflammation [23]. Not surprisingly, phospholipids were also the most correlated lipid classes with liver and kidney injury. Finally, the changes in metabolite and lipid abundance could be leveraged as a predictor of radiation injury with high accuracy. Another striking observation of this study was the accumulation of long-chain acylcarnitines (LCACs), including sterearoylcarnitine (C18:0) and linoleycarnitine (C18:2) across different tissue types (heart, lung, jejunum, spleen, liver, and kidney) in the surviving and non-surviving NHPs. Accumulation of this class of metabolites has been reported to cause hypoxia which is especially relevant in vital organs like the heart which derives 50–70% of its energy demands from the oxidation of fatty acids [24]. Mitochondrial oxidation of fatty acids requires these molecules to be esterified with carnitine to form acylcarnitines that is able to cross the mitochondrial membrane, which is otherwise impermeable to long-chain fatty acids [25]. It is well known that dysregulation in acylcarnitines and free fatty acids can cause disrupted mitochondrial function leading to radiation-induced tissue damage [26]. Additionally, given the pro-inflammatory properties of LCACs [27], it is reasonable to posit that radiation-induced tissue necrosis may mobilize elevated levels of LCACs in response to the cellular stress from radiation. We also observed a decrease in fatty acid amides including palmitoleamide, oleamide, stearamide, and 13-docasenamide across all five tissue types in the survivors and non-survivors. These metabolites are structurally analogous to endocannabinoids and have been reported to exert strong anti-inflammatory effects in the heart and brain and the endogenous levels are regulated by the enzyme fatty acid amine hydrolase (FAAH), which in turn has been shown to be a key regulator of endocannabinoid-induced myocardial tissue injury [28,29]. It is noteworthy that FAAH is predominantly present in the mitochondria and compromised functioning of this enzyme will significantly alter the lipid pools within the tissue [30]. Although preliminary, our results are indicative of radiation-induced dysregulated mitochondrial energetics, and it would be worthwhile to test this hypothesis in future studies.

Currently, there is a dearth of understanding about tissue-specific injury caused by exposure to ionizing radiation. Hence, the focus of this study was to examine tissue-specific changes in the survivor and non-survivor population. Taken together, these findings show that while radiation has tissue-specific metabolic alterations, there are also some underlying patterns of metabolite abundance that may help define a common pattern of radiation-induced tissue injury. Future studies will be needed to determine if these changes can be corroborated in minimally invasive matrices to stratify individuals at risk of radiation toxicities.

While this study underscores the value of molecular phenotyping technologies for the development of high-accuracy biomarkers of radiation effects, one of the shortcomings of this study is the lack of delineation of longitudinal changes in metabolism that would provide insights into how these changes could be detected earlier and leveraged for early detection and potential mitigation of radiation-induced tissue/organ damage. Future studies will focus on addressing this gap by identifying metabolic biomarker profiles at earlier time points that could be used to predict delayed radiation injury in late responding tissues including kidney, heart, lung, and brain.

## 4. Materials and Methods

### 4.1. Animals and Animal Care

Forty naïve rhesus macaques (*Macaca mulatta*, Chinese substrain, 36 males and 4 females) 3–5 years of age, weighing 4.15 to 7 kg, were obtained from the National Institutes of Health Animal Center (NIHAC, Poolesville, MD, USA) and maintained in a facility accredited by the Association for Assessment and Accreditation of Laboratory Animal Care (AAALAC)-International. Animals were quarantined for six weeks prior to the initiation of the experiment. Animal housing, health monitoring, care, and enrichment during the experimental period have been described earlier [31]. Animals were fed primate diet (Teklad T.2050 diet; Harlan^®^ Laboratories Inc., Madison, WI, USA) twice daily with at least six hours between feedings (animals were fed four biscuits each at 07:00 AM and 02:00 PM) and received drinking water *ad libitum*. Due to study-specific reasons, paired housing was not possible during the experiment. The animals were housed individually, but they were able to see and touch conspecifics through the cage divider. This also eliminated the chance of conflict injuries that could have been caused by pair-housing. Animals that are irradiated are more prone to infection as their natural immunity is suppressed. All procedures involving animals were approved by the Armed Forces Radiobiology Research Institute Institutional Animal Care and Use Committee (IACUC) and Department of Defense Animal Care and Use Review Office (ACURO). This study was carried out in strict accordance with the recommendations in the *Guide for the Care and Use of Laboratory Animals of the National Institutes of Health* [32].

### 4.2. Radiation Exposure

Food was withheld from each animal approximately 12–18 h prior to radiation exposure to minimize the occurrence of radiation-induced vomiting. To deliver the precise dose, NHPs’ abdominal widths were measured with digital calipers. Approximately 30–45 min prior to irradiation, NHPs were administered 10–15 mg/kg of ketamine hydrochloride intramuscularly for sedation, then placed in custom-made Plexiglas irradiation boxes and secured in a seated position. Two NHPs were placed on the irradiation platform facing away from each other and an exposure dose of 7.2 Gy (LD_70/60_ without supportive care) ^60^Cocobalt-60 γ-radiation at a dose rate of 0.6 Gy/min from both sides of the core of the abdomen (bilateral, simultaneous exposure). The radiation field in the area of the NHP location was uniform within ±1.5%. Animals were observed throughout the irradiation procedure via in-room cameras. Following irradiation, animals were returned to the transport cart and to their cages in the housing area and monitored for recovery from the procedure. AFRRI’s dosimetry for photons is based on the alanine/EPR (electron paramagnetic resonance) dosimetry system. This is one of the most precise dosimetry techniques at present, which is used, in particular, by national standard laboratories for the most critical measurements and calibrations. Thus, it is one of the very few methods that are used in regular worldwide inter-comparisons of the national standards of Gray. All other details on NHP radiation-exposure are described earlier [31].

### 4.3. Tissue Sample Collection

The heart, lung, spleen, jejunum, liver, and kidney were collected at various days’ post-irradiation in liquid nitrogen or dry ice immediately after euthanasia during necropsy. Tissue samples were stored at −70 °C until shipped on dry ice to Georgetown University Medical Center (Washington, DC, USA).

### 4.4. Sample Preparation and LC-MS Analyses

All tissue types were prepared uniformly for metabolomic analysis. Metabolite extraction was performed by adding 150 μL of 40% isopropanol (IPA) + 25% methanol + 35% water containing internal standards to NHP tissue. Samples were then homogenized on ice and then 150 μL of 100% acetonitrile (ACN) as added. Samples were vortexed and incubated on ice for 20 min to facilitate protein precipitation. Vials were centrifuged at 13,000 rpm at 4 °C for 20 min. The supernatant was transferred to fresh vials for UPLC-ESI-Q-TOF-MS analysis. Protein quantitation was carried out on the remaining tissue pellet using a Bradford assay.

For metabolomic analysis, 2 μL of each sample was injected onto a reverse-phase 50 × 2.1 mm Acquity 1.7-μm BEH C18 column at a temperature 40 °C (Waters Corp, Milford, MA, USA) using an Acquity UPLC system (Waters) with a gradient mobile phase consisting of 100% water containing 0.1% formic acid (Solvent A) and 100% ACN containing 0.1% formic acid (Solvent B) and 90% IPA + 10% ACN containing 0.1% formic acid (Solvent C), and resolved for 13 min at a flow rate of 0.4 mL/min. The gradient started with 95% A and 5% B for 0.5 min with a ramp of curve 6. At 8 min, the gradient reached 2% A and 98% B. From 8 to 9 min, the gradient shifted to 0% A and 2% B and 98% C for 1.5 min. From 10.5 to 11.5 min, the gradient shifted to 50% A and 50% B. From 11.5 to 12.5 min, it shifted to 95% A and 5% B (initial condition).

The column eluent was introduced directly into the mass spectrometer by electro-spray. Mass spectrometry was performed on a Q-TOF MS (Xevo G2 QTOF MS, Waters Corporation, Milford, MA, USA), operating in either negative-ion (ESI−) or positive-ion (ESI+) electrospray ionization mode with a capillary voltage of 3 kV for positive mode and 1.5 kV for negative mode and a sampling cone voltage of 30 V in both negative and positive modes. The extraction cone was 3.0. The desolvation gas flow was set to 1000 L/h and the temperature was set to 500 °C. The cone gas flow was 25 L/h, and the source temperature was 120 °C. The accurate mass was maintained by the introduction of the LockSpray interface of Leucine Enkephalin (556.2771 [M + H]^+^ or 554.2615 [M − H]^−^) at a concentration of 2 ng/μL in 50% aqueous ACN and a rate of 5 μL/min. Data were acquired in centroid mode from 50 to 1200 m/z in MS scanning. Pooled QC (quality control samples) were run throughout the batch to monitor data reproducibility.

### 4.5. Statistical Analyses

Centroided and integrated mass spectrometry data from the UPLC-TOFMS were preprocessed using XCMS software (Scripps Research Institute, La Jolla, CA, USA) to generate a data matrix containing ion intensities, mass to charge (*m*/*z*), and retention time values. The data were normalized to the intensities of the internal standards and protein quantification. Multivariate statistics were performed using R scripts developed in-house [33] with Pareto scaling and log-transformation used for data normalization. ANOVA and Student’s t-test comparisons were used to identify significantly dysregulated metabolites (based on *m*/*z* values) between comparative groups and further corrected using the Benjamini–Hochberg (FDR) multiple testing correction method and then were subjected to a database search for the identification and biological relevance using Metlin [34] CEU Mass Mediator [35] and HMDB [36]. The identity of these significantly dysregulated metabolites was confirmed using tandem mass spectrometry (Supplementary Table S2). Additionally, the identity of lipids was confirmed by using the SIMPLIPID software V6.01 (Premier Biosoft, USA), by fragmentation pattern matching. Figures were generated using custom R scripts. The ROC curves were calculated using the pROC package [37], and the survival curve was created using the survival package [38].

## Supplementary Materials

The following are available online at https://www.mdpi.com/1422-0067/20/13/3360/s1, Supplementary Table S1. ANOVA analysis of significantly dysregulated metabolites across tissue types. Supplementary Table S2. Collision induced dissociation (CID) fragmentation results for significantly dysregulated metabolites in response to exposure to ionizing radiation.
