# Supplementary material for: Metabolomic Studies of Tissue Injury in Nonhuman Primates Exposed to Gamma-Radiation
Source: Int J Mol Sci. 2019 Jul 9;20(13):3360. doi: 10.3390/ijms20133360 (PMC6651211; doi:10.3390/ijms20133360)
Supplement: Supplementary file 1 [file ijms-20-03360-s001.zip › Supplementary Table 1.pdf]

Supplementary table 1

Metabolite	m/z	Mass Error(ppm)	Retention Time(min)	Mode	CID Fragments
3-Hydroxy-5-phenylpentanoic acid	193.0875	3	3.87	NEG	68.9931
Xanthurenic Acid	204.0294	4	1.2	NEG	186.0191
N-lactoyl-Phenylalanine	236.0933	3	3.74	NEG	44.9982
Abu-Gly-OH	267.0634	4	0.43	NEG	136.0506, 108.0213, 92.0251
PE(16:0/0:0)	452.2762	5	6.63	NEG	255.23, 196.03, 152.99
1-hexadecanoyl-sn—glycerol-3-phosphor-D-myo-inositol	571.2831	10	6.64	NEG	255.23, 152.99
6-O-(1-O-stearyol-sn-glycerol-3-phosphono)-1D-myo-inositol	599.317	5	7.89	NEG	283.26, 241.01, 152.99
PI(20:4/0:0)	619.2835	9	6.21	NEG	315.04, 241.01, 152.99
PE(18:2/16:1)	712.4897	4	9.8	NEG	279.21, 253.21
PE(16:0/18:2)	714.5055	3	9.9	NEG	452.27, 279.23, 255.23, 196.03, 152.99, 140.01
PE(16:0/18:1)	716.518	8	9.94	NEG	281.24, 255.23, 196.03, 140.01
PE(P-16:0/20:4)	722.5091	5	9.9	NEG	418.27, 303.23, 196.03, 152.99, 140.01
PA(P-18:0/18:0)	723.5134	8	9.91	NEG	152.99
PE(18:2/18:2)	738.5053	4	9.85	NEG	720.49, 279.23
PE(18:0/18:2)	742.5366	4	9.93	NEG	480.30, 462.29, 279.23, 196.03, 152.99, 140.01
LBPA(16:0/18:1)	747.5188	1	9.89	NEG	729.51, 417.24, 391.22, 281.24, 255.23, 152.99
PE(P-18:0/20:4)	750.5384	8	9.97	NEG	732.56, 464.31, 446.30, 303.23, 196.03, 140.01
PE(20:4/18:2)	762.5053	3	9.8	NEG	303.23, 279.23
PE(18:2/20:3)	764.519	6	9.85	NEG	746.51, 728.52, 721.49, 305.24, 279.23, 196.03, 152.99, 140.01
PE(18:0/20:4)	766.5364	4	9.93	NEG	748.52, 730.52, 723.51, 480.30, 303.23, 283.26, 196.03, 152.99, 140.01
PG(18:1/18:2)	771.5216	4	9.89	NEG	281.24, 279.23
PE(22:6/P-18:1)	772.5271	2	9.88	NEG	462.2990, 444.2884, 327.2330, 283.2431, 196.0380, 104.0118, 78.9591
PG(18:0/18:2)	773.5313	3	9.89	NEG	737.53, 699.50, 437.28, 419.25, 283.26, 279.23, 152.99
PA(P-18:0/22:2)	775.5368	6	9.9	NEG	739.51, 303.23, 152.99
PE(20:3/20:4)	788.5233	0	9.83	NEG	770.50, 752.51, 305.24, 303.23
PC(P-16:0/20:3)	802.5569	6	9.88	NEG	766.53, 752.55, 464.31, 305.24, 168.04
PA(22:0/22:6)	803.5604	1	9.88	NEG	767.54, 687.54, 327.23, 152.99, 78.95
PS(18:0/20:4)	810.5223	8	12.02	NEG	419.25, 152.99
PG(20:3/20:4)	819.5168	2	9.81	NEG	305.24, 303.23
PC(20:4/P-18:1)	826.5567	6	9.81	NEG	790.53, 776.54, 303.23, 168.04
PS(18:1/21:0)	830.5869	6	9.95	NEG	812.57, 794.56, 743.54, 725.52, 417.24, 281.24, 152.99
PC(P-20:0/18:2)	832.5989	0	9.98	NEG	814.58, 796.58, 279.23
PI(16:0/18:1)	835.5288	9	9.98	NEG	281.24, 255.23, 241.01, 223.00, 152.99
PC(O-20:1/20:4)	856.5962	4	9.94	NEG	820.58, 806.58, 717.52, 699.50, 303.23
PI(18:2/18:1)	859.5271	8	9.94	NEG	281.24, 279.23, 241.01, 223.00, 152.99
PI(18:2/18:0)	861.5459	5	9.99	NEG	699.50, 599.31, 581.30, 283.26, 279.23, 241.01, 223.00, 152.99
PG(22:6/22:4)	869.532	2	9.8	NEG	851.56, 795.51, 715.51, 331.26, 327.23
PI(P-18:0/17:0)	871.5432	5	9.92	NEG	303.23, 152.99
PI(18:0/20:4)	885.5466	4	9.96	NEG	599.31, 581.30, 303.23, 283.26, 241.01, 223.00, 152.99
Choline	104.108	9	0.38	POS	60.0847
Glutamate	148.0608	2	0.4	POS	130.0545, 85.0285, 84.0446
Xanthine	153.0414	5	0.43	POS	136.0140, 135.0300, 110.0348, 82.0400, 55.0291
Carnitine	162.1136	6	0.4	POS	103.0394, 102.0920, 85.0291, 60.0819,
1,2-Dihydronaphthalene-1,2-diol	163.0763	6	4.33	POS	115.0542, 107.0491
Phenylpyruvic acid	165.0555	6	0.42	POS	91.0553
Phenylalanine	166.0852	6	0.63	POS	149.0587, 131.0484, 120.0806, 107.0485, 103.0537, 93.0702, 79.0548, 77.0392
Valero-1,5-lactam	172.116	5	8.71	POS	65.0403, 41.0379
Choline Sulfate	184.0646	4	0.37	POS	166.0530, 124.9900, 104.1070, 86.0964, 84.9641, 60.0808
10-Oxo-8-decenoic acid	185.1158	7	3.15	POS	69.0699
3-amino-2-naphthoic acid	188.071	2	1.17	POS	170.0601, 142.0650, 115.0540
N6,N6,N6-Trimethyl-L-Lysine	189.1616	10	0.36	POS	130.0863, 84.0808, 60.0808
Acetylcarnitine	204.1239	4	0.41	POS	145.0488, 85.0275
(+-)-propionylcarnitine	218.1403	7	0.45	POS	218.1386, 159.0642, 85.0288, 60.0815, 44.0483
6,8,10,12-pentadecatetraenal	219.1753	4	6.66	POS	107.0860, 91.0542
pro ile	229.1557	5	0.42	POS	70.0645
isobutrylcarnitine	232.1548	1	0.84	POS	86.0964
2-methylbutroylcarnitine	246.1704	1	2.02	POS	187.0963, 85.0288, 60.0814, 57.0702
palmitoleamide	254.2454	10	5.18	POS	151.1480, 69.0699
Palmitic amide	256.2637	0	7.8	POS	186.1829, 109.1007, 102.0914, 88.0758, 81.0694, 69.0691
Glycerophosphocholine	258.1096	2	0.39	POS	184.0724, 166.0607, 124.9999, 104.1074, 86.0977
3-carboxy-4-methyl-5-pentyl-2-furanpropanoic acid	269.1366	7	3.31	POS	181.0495
C16 Sphinganine	274.2745	2	5.04	POS	256.26
Gamma-glutamylglutamic acid	277.1016	5	0.4	POS	259.0925, 242.0659, 148.0604, 130.0499, 102.0550, 84.0444
Oleamide	282.2798	2	9.99	POS	149.1318, 121.0980, 81.0747, 69.0684, 57.0711
Stearamide	284.295	1	8.55	POS	102.0918, 97.1003, 88.0755, 83.0854
12,13 EpOME	297.2406	6	7.39	POS	69.0699, 57.0699
Sphinganine	302.3051	1	5.72	POS	284.2929, 43.0552
Eicosapentaenoic acid	303.2305	4	7.53	POS	097, 243.2062, 215.1327, 189.1625, 145.1045, 135.1170, 121.1007, 119.0859, 109.0990, 105.0646, 91.0546, 79.0542, 71.0466, 67.0557, 55.0554
3-oxo-nonadecanoic acid	313.2739	0	8.05	POS	221.2260, 85.1012, 71.0855, 69.0699, 57.0699, 55.0542, 43.0542
4,7,10,13,16,19-docosahexanoic acid	329.247	1	7.82	POS	296, 187.1447, 181.1012, 171.1152, 159.1156, 149.1296, 147.1158, 145.1006, 143.0850, 131.0852, 129.0694, 119.0854, 117.0698, 105.0701, 95.0862, 93.0703, 91.0545, 81.0704, 67.0548
13-Docosenamide	338.342	0	9.11	POS	1982, 163.1446, 153.1270, 149.1307, 139.1104, 135.1148, 121.1000, 109.1009, 97.1004, 95.0854, 93.0704, 83.0860, 81.0701, 79.0548, 69.0705, 67.0530
2-oxo-heneicosanoic acid	341.3048	1	8.72	POS	71.0855, 57.0699
Adenosine 3'-monophosphate	348.0718	4	0.4	POS	136.0616, 119.0351, 97.0286
O-Arachidonoyl Glycidol (2AG)	361.2727	2	6.2	POS	203.1785, 171.1154, 157.1008, 145.1001, 135.1159, 133.1006, 121.1008, 119.0852, 105.0700, 93.0700, 91.0544, 81.0701, 79.0545, 67.0546, 43.0551
Linoleyl carnitine (C18:2)	424.3398	5	6.38	POS	144.1020, 97.1012, 85.0284
C18:1	426.3575	2	6.73	POS	144.1020, 85.0284
Sterearoylcarnitine (C18:0)	428.3734	0	7.13	POS	267.2680, 144.1020, 85.0284, 43.0542
PC(13:0/0:0)	454.293	0	6.59	POS	436.28, 258.11, 184.07, 166.06, 104.10, 86.09
PC(P-15:0/0:0)	466.3287	1	7.55	POS	448.31, 184.07, 104.10
PC(15:0/0:0)	482.3248	1	7.35	POS	258.11, 184.07, 166.06, 104.10, 86.09
LysoPAF C-16	482.3601	0	6.79	POS	184.0738, 166.0594, 124.9992, 104.1066, 86.0968, 60.0803
PC(16:0/0:0)	496.3402	1	6.61	POS	478.32, 313.27, 258.11, 184.07, 166.06, 104.10, 86.09
PC(P-18:1/0:0)	506.3562	8	7.37	POS	421.27, 341.30, 184.07, 166.06, 104.10, 86.09
FAHFA(16:1/5-O-16:0)	509.4552	2	10.21	POS	237.22, 163.14, 149.13, 135.11, 121.10, 107.08
PC(O-16:0/1:0)	510.3552	0	6.96	POS	184.07, 104.10
Lyso-PAF-C-18	510.3887	6	7.59	POS	184.0728, 124.9993, 104.1068, 86.0967, 43.0542
PC(18:3/0:0)	518.3217	0	6.61	POS	433.23, 258.11, 184.07, 166.06, 104.10, 86.09
PC(18:2/0:0)	520.3402	0	6.23	POS	337.27, 240.10, 184.07, 166.06, 104.10, 86.09
PC(18:0/0:0)	524.3715	1	7.37	POS	506.36, 421.27, 341.30, 258.11, 184.07, 166.06, 104.10, 86.09
PC(20:5/0:0)	542.3214	2	6.21	POS	184.07, 166.06, 104.10, 86.09
PC(20:4/0:0)	544.3395	3	6.23	POS	526.34, 361.27, 240.10, 184.07, 166.06, 104.10, 86.09
PC(20:3/0:0)	546.3541	1	7.36	POS	461.26, 443.23, 258.11, 184.07, 166.06, 104.10, 86.09
PC(O-16:0/4:0)	552.3978	8	8.12	POS	184.07
PC(20:4/0:0)	566.3214	2	6.22	POS	184.07
PC(22:6/0:0)	568.3384	2	6.22	POS	550.34, 483.24, 385.27, 240.10, 184.07, 166.06, 104.10, 86.09
PC(O-18:2/2:0)	582.3687		6.62	POS	184.07, 104.10
6-O-(1-O-stearoyl-sn-glycero-3-phosphono)-1D-myo-inositol	601.3323	4	7.92	POS	565.31, 341.30
DG(18:0/20:4/0:0)	645.5446	1	9.99	POS	361.27, 341.30, 287.27, 283.27, 267.26
SM(d18:1/14:0)	675.5417	3	9.66	POS	184.07, 86.09
PC(12:0/16:0)	678.5125	8	9.7	POS	478.32, 184.08, 166.06, 104.10, 86.09
SM(d16:1/17:0)	689.5581	2	9.79	POS	630.51, 184.08, 166.06, 104.10, 86.09
SM(d18:1/14:0)	697.5213	3	9.65	POS	638.45, 514.46, 184.07, 86.09
PC(O-16:0/13:0)	700.5312	9	9.94	POS	504.34, 184.08
SM(d16:1/18:1)	701.5571	3	9.7	POS	598.48, 184.07, 86.09
SM(d18:1/16:0)	703.574	1	9.88	POS	618.51, 282.27, 184.08, 166.06, 104.10, 86.09
SM(d18:0/16:0)	705.5803	14	9.89	POS	184.08, 166.06, 104.10, 86.09
Butyl 4'-O-butanoyl-6-O-hexadecanoyl-neohesperidoside	713.4415	5	6.59	POS	625.39, 497.34, 479.33
PC(13:0/18:2)	716.5269	6	9.9	POS	502.32, 184.08, 166.06, 104.10, 86.09
PC(P-16:0/15:1)	724.5272	3	9.91	POS	502.32, 184.08
PC(P-16:0/15:0)	726.538	4	9.92	POS	502.32, 184.08
PC(O-16:0/15:0)	728.559	4	9.98	POS	502.34, 184.08
PC(14:0/18:2)	730.5427	6	9.75	POS	520.34, 502.32, 184.08, 166.06, 104.10, 86.09
PC(16:0/16:1)	732.5575	5	9.85	POS	478.32, 239.23, 184.08, 166.06, 104.10, 86.09
PC(14:0/18:0)	734.569	1	9.93	POS	716.52, 524.36, 506.35, 267.26, 184.08, 166.06, 104.10, 86.09
PC(13:0/20:4)	740.5252	4	9.83	POS	526.32, 184.08, 166.06, 104.10, 86.09
PC(P-16:0/17:2)	750.5533	16	9.93	POS	714.51, 502.32, 184.08
PC(14:0/20:5)	752.5144	10	6.59	POS	184.07, 166.06, 104.10, 86.09
PC(O-16:0/17:2)	752.5585	3	9.96	POS	504.34, 184.08
PC(16:1/18:2)	756.5544	1	9.75	POS	573.48, 520.34, 502.32, 184.08, 166.06, 104.10, 86.09
PC(16:0/18:2)	758.5693	0	9.85	POS	722.55, 575.50, 520.33, 502.32, 496.33, 478.32, 184.08, 166.06, 104.10, 86.09
PC(18:0/16:1)	760.583	3	9.91	POS	742.56, 577.51, 524.36, 506.35, 267.26, 184.08, 166.06, 104.10, 86.09
PC(17:2/18:0)	772.5892	5	9.91	POS	589.51, 524.36, 506.35, 267.26, 184.08, 166.06, 104.10, 86.09
PC(O-15:0/20:4)	776.5588	3	9.91	POS	184.08
PC(P-18:0/17:2)	778.5659	8	9.9	POS	760.58, 576.50, 184.08
PC(16:0/20:4)	782.5694	0	9.8	POS	746.56, 599.50, 526.32, 496.33, 478.32, 239.23, 184.08, 166.06, 104.10, 86.09
PC(16:0/20:3)	784.5826	3	9.84	POS	766.53, 663.45, 601.51, 583.50, 496.33, 478.32, 239.23, 184.08, 166.06, 104.10, 86.09
PC(18:0/18:2)	786.5995	2	9.92	POS	768.58, 750.54, 665.45, 603.53, 585.52, 524.36, 506.35, 502.32, 267.26, 263.23, 184.08, 166.06, 104.10, 86.09
PC(17:1/19:0)	788.6103	8	9.95	POS	788.60, 770.59, 667.52, 605.54, 587.54, 281.29, 184.08
PC(O-16:0/22:6)	792.5825	10	9.84	POS	184.08, 166.06, 104.10, 86.09
PC(20:3/P-18:1)	794.5997	8	9.89	POS	611.53, 506.35, 184.08, 166.06, 104.10, 86.09
PC(18:2/20:5)	804.5527	1	9.74	POS	768.55, 719.46, 621.48, 603.50, 520.34, 502.32, 184.08, 166.06, 104.10, 86.09
PC(18:2/20:4)	806.5694	0	9.75	POS	788.57, 770.56, 623.50, 605.51, 526.32, 520.34, 502.32, 184.08, 166.06, 104.10, 86.09
PC(16:0/22:5)	808.5832	2	9.8	POS	790.56, 723.51, 625.51, 607.50, 496.33, 478.32, 239.23, 184.08, 166.06, 104.10, 86.09
PC(16:0/22:4)	810.5992	2	9.89	POS	792.58, 774.59, 725.53, 627.53, 609.52, 496.33, 478.32, 184.08, 166.06, 104.10, 86.09
PC(18:0/20:3)	812.6134	4	9.92	POS	794.60, 776.56, 727.54, 629.54, 611.53, 524.36, 506.35, 267.26, 184.08, 166.06, 104.10, 86.09
PC(P-18:0/22:6)	818.602	5	9.83	POS	635.54, 184.08, 166.06, 104.10, 86.09
PC(18:0/19:1)	824.6083	7	9.84	POS	806.57, 788.57, 738.50, 526.32, 184.08
PC(20:4/20:5)	828.5522	2	9.73	POS	792.55, 743.46, 645.48, 526.33, 184.08, 166.06, 104.10, 86.09
PC(18:2/22:6)	830.5684	1	9.66	POS	812.57, 812.52, 647.50, 629.51, 184.07, 86.09
PC(18:1/22:5)	834.5989	2	9.84	POS	798.59, 749.53, 651.53, 522.35, 504.34, 184.08, 166.0, 104.10, 86.09
PS(16:1/22:2)	836.5411	0	12	POS	651.53, 207.99
PC(18:0/22:4)	838.624	10	9.93	POS	802.62, 753.56, 655.56, 637.55, 524.36, 506.35, 267.26, 184.08, 166.06, 104.10, 86.09
PC(19:1/22:6)	846.5905	12	9.74	POS	810.56, 681.55, 184.07, 86.09
PC(19:0/22:6)	848.6165	0	9.78	POS	812.55, 763.56, 550.32, 184.08, 166.06, 104.10, 86.09
PC(20:4/22:6)	854.5688	1	9.62	POS	769.47, 671.50, 550.32, 526.32, 184.08, 166.06, 104.10, 86.09
PC(20:3/22:6)	856.5829	3	9.67	POS	771.48, 753.52, 673.51, 184.07, 86.09
PC(O-20:0/22:2)	856.72	5	10.14	POS	735.57, 184.07
PC(18:0/22:4)	860.6189	10	9.79	POS	526.33, 184.07
PC(19:0/22:6)	870.5921	0	9.59	POS	852.55, 184.07
PC(22:6/22:6)	878.5716	3	9.54	POS	184.07
