# Supplementary material for: Metabolomic Studies of Tissue Injury in Nonhuman Primates Exposed to Gamma-Radiation
Source: Int J Mol Sci. 2019 Jul 9;20(13):3360. doi: 10.3390/ijms20133360 (PMC6651211; doi:10.3390/ijms20133360)
Supplement: Supplementary file 1 [file ijms-20-03360-s001.zip › Supplementary Table 2. Lipids.pdf]

Supplementary table 2. Lipids

*ANOVA results portray FDR adjusted p-value *Binary comparison results portray FDR adjusted p-valuse (fold change)

Metabolites	Jejunum				Lung				Kidney				Liver				Spleen				Heart			
	ANOVA	HvS	HvNS	SvNS	ANOVA	HvS	HvNS	SvNS	ANOVA	HvS	HvNS	SvNS	ANOVA	HvS	HvNS	SvNS	ANOVA	HvS	HvNS	SvNS	ANOVA	HvS	HvNS	SvNS
PC(22:6/0:0)	0.019			0.032 (↑2.91)							0.047(↑1.74)		0.001		0.002(↑10.93)	0.025(↑2.46)								
PC(14:0/18:0)	0.026								0.009	0.017(↓0.59)	0.011(↓0.57)		0.02			0.007(↑1.78)								
SM(d18:1/16:0)					0.046				0.005	0.017(↓0.45)	0.003(↓0.47)		0.015			0.026(↑1.55)								
6-O-(1-O-stearoyl-sn-glycero-3-phosphono)-1D-myo-inositol									0.009	0.017(↓0.59)	0.018(↓0.61)		0.048								0.034			
PC(22:6/22:6)	0.019			0.035 (↑1.71)									0.024			0.031(↑1.72)	0.011			0.011(↑1.92)				
PC(18:2/20:4)					0.046								0.005			0.004(↑1.66)	0.027			0.016(↑1.99)				
PC(13:0/0:0)									0.038	0.022(↓0.41)			0.005			0.004(↑7.2)								
PC(P-15:0/0:0)									0.033	0.017(↓0.43)			0.017			0.011(↑3.31)								
PC(15:0/0:0)									0.005	0.017(↓0.29)	0.014(↓0.32)		0.014			0.01(↑4.26)								
PC(O-18:2/2:0)										0.031(↓0.6)			0.042			0.016(↑1.71)								
SM(d18:1/14:0)									0.027	0.017(↓0.64)														
PC(13:0/18:2)									0.026	0.025(↓0.61)	0.015(↓0.57)		0.048			0.046(↑1.62)								
PC(P-16:0/15:1)									0.005	0.017(↓0.56)	0.003(↓0.49)					0.046(↑1.51)								
PC(13:0/20:4)									0.011	0.018(↓0.58)	0.007(↓0.54)		0.014			0.023(↑1.58)								
PC(14:0/20:5)									0.049	0.03(↓0.59)						0.043(↑1.37)								
PC(16:0/18:2)									0.01	0.025(↓0.67)	0.004(↓0.58)		0.024			0.01(↑1.54)								
PC(18:0/16:1)									0.019	0.017(↓0.66)	0.016(↓0.66)					0.044(↑1.36)								
PC(16:0/20:4)									0.016		0.005(↓0.66)		0.019			0.01(↑1.38)								
PC(16:0/22:5)									0.041		0.048(↓0.73)					0.044(↑1.24)								
PC(16:0/22:4)									0.023	0.032(↓0.71)	0.016(↓0.64)					0.05(↑1.23)								
PC(20:4/20:5)											0.033(↓0.72)		0.038			0.016(↑1.39)								
PC(18:1/22:5)										0.05(↓0.75)						0.031(↑1.41)								
PS(16:1/22:2)									0.014	0.025(↓0.69)	0.008(↓0.6)		0.013			0.006(↑1.63)								
SM(d18:0/16:0)					0.046				0.005	0.017(↓0.5)	0.004(↓0.5)													
PC(O-16:0/15:0)									0.008	0.017(↓0.64)	0.006(↓0.6)						0.027			0.02(↓0.66)				
PC(14:0/18:2)											0.021(↓0.69)									0.026(↓0.68)				
PC(16:0/16:1)									0.008		0.003(↓0.52)						0.043			0.014(↓0.55)				
PC(20:4/0:0)													0.005		0.006(↑6.29)	0.046(↑1.99)								
PC(19:0/22:6)																0.048(↑1.46)								
PE(20:3/20:4)	0.0002	0.005(↓0.57)	0.001(↓0.45)				0.045(↓0.49)		0.033	0.023(↓0.65)	0.031(↓0.65)													
PE(16:0/0:0)									0.046	0.027(↓0.4)			0.009			0.014(↑5.89)								
PE(18:0/18:2)	0.038		0.02(↓0.68)						0.004	0.006(↓0.58)	0.01(↓0.56)													
LBPA(16:0/18:1)	0.008	0.024(↓0.62)	0.012(↓0.54)						0.004	0.005(↓0.51)	0.016(↓0.56)													
PC(P-16:0/20:3)			0.012(↓0.71)						0.01	0.013(↓0.63)	0.01(↓0.58)													
PA(22:0/22:6)	0.034		0.011(↓0.7)						0.011	0.015(↓0.63)	0.01(↓0.59)													
PS(18:0/20:4)		0.001(↓0.57)	0.001(↓0.47)						0.004	0.019(↓0.6)	0.01(↓0.48)													
PS(18:1/21:0)	0.015		0.009(↓0.7)						0.004	0.005(↓0.58)	0.01(↓0.59)													
PC(P-20:0/18:2)	0.011	0.017(↓0.72)	0.013(↓0.74)						0.007	0.005(↓0.59)	0.024(↓0.65)													
PI(16:0/18:1)	0.008	0.025(↓0.57)	0.012(↓0.58)						0.027	0.018(↓0.6)	0.029(↓0.6)													
PI(18:2/18:1)	0.024		0.022(↓0.56)						0.004	0.008(↓0.52)	0.01(↓0.41)													
PI(18:2/18:0)	0.001	0.02(↓0.55)	0.002(↓0.44)						0.013	0.007(↓0.56)	0.048(↓0.66)													
PI(P-18:0/17:0	0.011		0.006(↓0.56)						0.008	0.016(↓0.6)	0.012(↓0.53)													
PI(18:0/20:4)	0.001	0.02(↓0.61)	0.003(↓0.48)						0.012	0.01(↓0.57)	0.017(↓0.56)													
PI(20:4/0:0)													0.0206		0.036 (↑2.39)									
PE(18:2/16:1)									0.0334		0.01 (↓0.45)													
PE(16:0/18:2)									0.0277	0.021 (↓0.6)	0.029 (↓0.61)													
PE(16:0/18:1)									0.0071	0.005 (↓0.54)	0.032 (↓0.65)													
PE(P-16:0/20:4)																								
PA(P-18:0/18:0)									0.0038	0.005 (↓0.56)	0.01 (↓0.53)													
PE(18:2/18:2)									0.0188	0.011 (↓0.59)	0.031 (↓0.62)													
PE(P-18:0/20:4)									0.0047	0.005 (↓0.64)	0.01 (↓0.58)													
PE(20:4/18:2)										0.035 (↓0.64)														
PE(18:2/20:3)									0.0144	0.012 (↓0.62)	0.018 (↓0.6)													
PE(18:0/20:4)									0.0052	0.006 (↓0.59)	0.01 (↓0.57)													
PG(18:1/18:2)									0.0338	0.024 (↓0.67)	0.028 (↓0.63)													
PE(22:6/P-18:1)									0.031	0.012 (↓0.64)	0.037 (↓0.65)													
PG(18:0/18:2)										0.044 (↓0.69)	0.047 (↓0.66)													
PA(P-18:0/22:2)									0.0123	0.011 (↓0.6)	0.015 (↓0.57)													
PG(20:3/20:4)	0.0071			0.04 (↑1.4)																				
PC(20:4/P-18:1)																								
PC(O-20:1/20:4)																								
PG(22:6/22:4)			0.032 (↓0.75)																					
LysoPAF C-16													0.0499											
PC(16:0/0:0)													0.003		0.02 (↑3.86)	0.004 (↑3.07)								
PC(P-18:1/0:0)													0.0181			0.011 (↑2.37)								
FAHFA(16:1/5-O-16:0)									0.0053	0.017 (↓0.56)	0.003 (↓0.47)													
PC(O-16:0/1:0)									0.0141															
Lyso-PAF-C-18									0.0349	0.049 (↓0.7)	0.038 (↓0.65)													
PC(18:3/0:0)													0.003		0.014 (↑3.6)	0.004 (↑2.68)								
PC(18:2/0:0)													0.005		0.008 (7.32)									
PC(18:0/0:0)													0.0194			0.012 (↑2.31)								
PC(20:5/0:0)													0.0025		0.003 (6.85)	0.047 (↑1.89)								
PC(20:3/0:0)													0.0252			0.015 (↑2.16)								
PC(O-16:0/4:0)									0.0053		0.003 (↓0.55)													
DG(18:0/20:4/0:0)									0.0053	0.017 (↓0.53)	0.003 (↓0.43)													
PC(12:0/16:0)													0.0221			0.004 (↑1.61)								
SM(d16:1/17:0)									0.0053	0.017 (↓0.45)	0.009 (↓0.51)													
PC(O-16:0/13:0)									0.006	0.017 (↓0.56)	0.005 (↓0.54)													
SM(d16:1/18:1)													0.0169			0.008 (↑1.53)								
PC(P-16:0/15:0)									0.0053	0.017 (↓0.57)	0.003 (↓0.5)													
PC(P-16:0/17:2)									0.0053	0.017 (↓0.48)	0.004 (↓0.41)													
PC(O-16:0/17:2)									0.0053	0.017 (↓0.62)	0.003 (↓0.5)													
PC(16:1/18:2)									0.0438		0.018 (↓0.66)													
PC(17:2/18:0)									0.0054		0.003 (↓0.56)													
PC(O-15:0/20:4)									0.0249		0.007 (↓0.61)													
PC(P-18:0/17:2)									0.0165		0.006 (↓0.58)													
PC(16:0/20:3)									0.0053		0.003 (↓0.59)													
PC(18:0/18:2)									0.0055	0.017 (↓0.63)	0.004 (↓0.6)													
PC(17:1/19:0)									0.0067	0.017 (↓0.63)	0.012 (↓0.66)													
PC(O-16:0/22:6)									0.024		0.008 (↓0.66)													
PC(20:3/P-18:1)									0.0044		0.003 (↓0.61)													
PC(18:2/20:5)									0.0074		0.004 (↓0.61)													
PC(18:0/20:3)									0.0053		0.004 (↓0.56)													
PC(P-18:0/22:6)									0.0053		0.003 (↓0.51)													
PC(18:0/19:1)													0.0432											
PC(18:2/22:6)									0.0391															
PC(18:0/22:4)											0.023 (↓0.67)													
PC(19:1/22:6)																0.025 (↑1.66)								
PC(20:4/22:6)																0.038 (↑1.46)								
PC(20:3/22:6)									0.0387															
PC(O-20:0/22:2)									0.0061	0.023 (↓0.45)	0.006 (↓0.37)													
PC(18:1/22:4)									0.0187	0.028 (↓0.66)	0.011 (↓0.6)													
