# Supplementary material for: Metabolomic Studies of Tissue Injury in Nonhuman Primates Exposed to Gamma-Radiation
Source: Int J Mol Sci. 2019 Jul 9;20(13):3360. doi: 10.3390/ijms20133360 (PMC6651211; doi:10.3390/ijms20133360)
Supplement: Supplementary file 1 [file ijms-20-03360-s001.zip › Supplementary Table 2. Metabolites.pdf]

Supplementary table 2. Metabolites

*ANOVA results portray FDR adjusted p-value *Binary cor

Metabolites	ANOVA
3-Hydroxy-5-phenylpentanoic acid	
Xanthurenic Acid	
N-lactoyl-Phenylalanine	
Abu-Gly-OH	
1-hexadecanoyl-sn—glycerol-3-phosphor-D-myo-inositol	
6-O-(1-O-stearyol-sn-glycerol-3-phosphono)-1D-myo-inositol	0.0205
Choline	
Glutamate	
Xanthine	
Carnitine	
1,2-Dihydronaphthalene-1,2-diol	
Phenylpyruvic acid	
Phenylalanine	
Valero-1,5-lactam	
Choline Sulfate	
10-Oxo-8-decenoic acid	
3-amino-2-naphthoic acid	
N6,N6,N6-Trimethyl-L-Lysine	0.0472
Acetylcarnitine	
(+-)-propionylcarnitine	
6,8,10,12-pentadecatetraenal	
pro ile	
isobutrylcarnitine	
2-methylbutroylcarnitine	
palmitoleamide	
Glycerophosphocholine	
3-carboxy-4-methyl-5-pentyl-2-furanpropanoic acid	
C16 Sphinganine	
Gamma-glutamylglutamic acid	
Oleamide	
Stearamide	
12,13 EpOME	
Palmitic amide	
Sphinganine	
Eicosapentaenoic acid	
3-oxo-nonadecanoic acid	
(4,7,10,13,16,19)-docosa-4,7,10,13,16,19-hexanoic acid	
13-Docosenamide	
2-oxo-heneicosanoic acid	
Adenosine 3'-monophosphate	
O-Arachidonoyl Glycidol (2AG)	

Linoleyl carnitine (C18:2)
C18:1
Sterearoylcarnitine (C18:0)
Butyl 4'-O-butanoyl-6-O-hexadecanoyl-neohesperidoside

nparison results portray FDR adjusted p-valuse (fold change)

Lung			Lung				
HvS	HvNS	SvNS	ANOVA	HvS	HvNS	SvNS	ANOVA
							0.0038
							0.0038
0.05 (↑3.09)							
0.026 (↓0.26)							
							0.0039
							0.0053
							0.0137
							0.0059
							0.0053
							0.0362
			0.0472				
							0.0337
							0.0091
							0.0053
							0.037
							0.0053
		0.032 (↑2.42)					
							0.0084
							0.0039
							0.0056
							0.0084
							0.0421
							0.0053
							0.0053
							0.0058
							0.0053
							0.0053
							0.0088
							0.0143
							0.0053

		0.0442

Kidney			Liver			
HvS	HvNS	SvNS	ANOVA	HvS	HvNS	SvNS
0.005 (↓0.55)	0.01 (↓0.51)					
			0.0153			
0.005 (↓0.56)	0.01 (↓0.5)					
0.038 (↓0.73)	0.041 (↓0.7)					
			0.0105			0.007 (↑1.72)
0.017 (↓0.45)	0.003 (↓0.35)					
			0.004			0.003 (↑2.07)
0.017 (↓0.6)	0.003 (↓0.5)					
0.046 (↑1.6)						
0.018 (↑1.66)	0.011 (↑1.78)					
0.018 (↓0.61)	0.004 (↓0.52)					
			0.0375			0.012 (↑1.96)
0.017 (↓0.58)	0.003 (↓0.51)					0.045 (↑1.34)
0.028 (↑1.44)	0.036 (↑1.53)		0.0064		0.049 (↑3)	0.044 (↑1.83)
			0.0051	0.02 (↓0.45)		0.023 (↑1.63)
						0.043 (↑1.8)
	0.035 (↑3.04)		0.0181			0.023 (↑3.96)
0.017 (↓0.61)	0.003 (↓0.51)					
			0.0058			0.006 (↑1.96)
			0.004			0.007 (↑5.88)
			0.0029		0.027 (↑6.09)	0.006 (↑4.08)
0.023 (↓0.65)	0.005 (↓0.57)					0.03 (↑1.39)
	0.003 (↑5.06)	0.021 (↑4.74)	0.0462			
0.017 (↓0.57)	0.005 (↓0.5)					0.046 (↑1.34)
0.026 (↓0.68)	0.005 (↓0.6)					0.037 (↑1.33)
	0.022 (↓0.74)		0.0161			0.046 (↑2.11)
0.017 (↓0.56)	0.003 (↓0.49)					0.048 (↑1.36)
0.017 (↓0.59)	0.003 (↓0.51)					0.044 (↑1.35)
0.018 (↓0.6)	0.004 (↓0.51)					
0.017 (↓0.57)	0.003 (↓0.5)					0.049 (↑1.33)
0.017 (↓0.6)	0.003 (↓0.52)					
			0.046			
	0.005 (↓0.53)					
			0.0042			0.006 (↑6.97)
	0.003 (↓0.54)		0.0423			0.034 (↑1.52)
0.017 (↓0.6)	0.003 (↓0.5)					
			0.009		0.027 (↑2.79)	0.043 (↑1.69)

0.03 (↓0.34)		0.015		0.03 (↑3.44)
		0.0218	0.049 (↑6.73)	
0.025 (↓0.47)		0.0047		0.006 (↑4.17)
0.037 (↓0.57)	0.047 (↓0.56)			0.029 (↑1.49)

Spleen				Heart			
ANOVA	HvS	HvNS	SvNS	ANOVA	HvS	HvNS	SvNS
				0.0227			
			0.039 (↓0.62)				
			0.043 (↓0.39)				
0.0397							
0.0174	.029 (↑4.99)						

0.042 (↑2.93)	
